# Inhibitory Effect of Thymoquinone on *Listeria monocytogenes* ATCC 19115 Biofilm Formation and Virulence Attributes Critical for Human Infection

**DOI:** 10.3389/fcimb.2019.00304

**Published:** 2019-08-27

**Authors:** Xin Miao, Huanhuan Liu, Yangyang Zheng, Du Guo, Chao Shi, Yunfeng Xu, Xiaodong Xia

**Affiliations:** ^1^College of Food Science and Engineering, Northwest A&F University, Yangling, China; ^2^College of Food and Bioengineering, Henan University of Science and Technology, Luoyang, China; ^3^Sino-US Joint Research Center for Food Safety, Northwest A&F University, Yangling, China

**Keywords:** *Listeria monocytogenes*, thymoquinone, biofilm formation, hemolysin, gene transcription

## Abstract

This study aimed to determine the antimicrobial activity of thymoquinone (TQ) against *Listeria monocytogenes*, and to examine its inhibitory effects on biofilm formation, motility, hemolysin production, and attachment-invasion of host cells. The minimum inhibitory concentrations (MICs) of TQ against eight different *L. monocytogenes* strains ranged from 6.25—12.50 μg/mL. Crystal violet staining showed that TQ clearly reduced biofilm biomass at sub-MICs in a dose-dependent manner. Scanning electron microscopy suggested that TQ inhibited biofilm formation on glass slides and induced an apparent collapse of biofilm architecture. At sub-MICs, TQ effectively inhibited the motility of *L. monocytogenes* ATCC 19115, and significantly impacted adhesion to and invasion of human colon adenocarcinoma cells as well as the secretion of listeriolysin O. Supporting these findings, real-time quantitative polymerase chain reaction analysis revealed that TQ down-regulated the transcription of genes associated with motility, biofilm formation, hemolysin secretion, and attachment-invasion in host cells. Overall, these findings confirm that TQ has the potential to be used to combat *L. monocytogenes* infection.

## Introduction

*Listeria monocytogenes* is a Gram-positive, non-spore-forming bacterium ubiquitously found in nature. It is a frequent cause of food-borne illness, with the most common sources of infection being processed foods, including raw milk products and ready-to-eat chilled foods (Farber and Peterkin, 1991; Allerberger and Wagner, 2010). Within the European Union, a statistically-significant increase in cases of listeriosis was noted between 2009 and 2015. Specifically, the number of confirmed human cases of listeriosis increased from 1,331 in 2009 to 2,206 in 2015 (EFSA, 2016). In general, the incidence of systemic listeriosis is much higher in susceptible populations, including pregnant women, the elderly, and immunocompromised individuals. Based on the susceptibility of the host, the severity of disease ranges from mild gastroenteritis to life-threatening infections such as septicemia, encephalitis, and meningitis (Zhu et al., 2017).

In addition, *L. monocytogenes* are invasive pathogens (Vazquez-Boland et al., 2001) and the interior of all cells that *L. monocytogenes* can penetrate, whether macrophages or non-professional phagocytic cells, such as epithelial cells (Jaradat and Bhunia, 2003), hepatocytes (Kanayama et al., 2015), endothelial cells (Greiffenberg et al., 1997; Das et al., 2001). *L. monocytogenes* can cross the placental barrier, blood-brain and intestinal barriers, leading to severe bacterial infections such as abortion of the fetus (Chen et al., 2017; Nowak et al., 2017). The gastrointestinal tract is the primary route of infection for *L. monocytogenes*, and adhesion to and invasion to intestinal epithelial cells, crossing the intestinal epithelial barrier is the first step (Drolia et al., 2018), and then subsequent translocation to distant organs are critical in establishing a systemic infection in a host (Jaradat and Bhunia, 2003; Drolia et al., 2018). Many virulence genes and proteins play an important role in the process of invading the host. *L. monocytogenes* invasion protein internalin A (Inl A) targets its basolateral receptor, E-cadherin, by host intrinsic mechanisms, the epithelial cell extrusion and goblet cell exocytosis allows its transcytosis across the intestinal barrier (Drolia and Bhunia, 2019). In addition, the gene *inl B* also encodes internalin involved in invasion of epithelial cells of *L. monocytogenes* (Dramsi et al., 1995). The broad-range phospholipase C (encoded by *plcA* and *plcB*, respectively) also play vital role in the process of *L. monocytogenes* escape from phagosome by formation of double-membrane vacuoles (Moors et al., 1999). Some researches focused on the sig B, a protein triggered during stress conditions and known to play an important role in regulating gene expression when there are major changes in the environment (Van Schaik et al., 2004). Listeriolysin O (LLO) is an oligomeric pore-forming toxin secreted by *L. monocytogenes*. Studies have shown that it is a member of the cholesterol-dependent cytolysin family (Kozorog et al., 2018; Lu et al., 2019). As a main virulence factor in *Listeria* pathogenesis, LLO has profound effects on the escape of *L. monocytogenes* from host-cell vacuoles (Portman et al., 2017). LLO production allows *L. monocytogenes* to rapidly escape from the phagolysosome, releasing the bacterium into the cytosol and bacteria spread (Seveau, 2014).

*L. monocytogenes* forms biofilms on both abiotic and biotic surfaces in food processing environments, a process that involves bacterial flagella (Lemon et al., 2007). Costerton et al. (1999) reported that bacteria found in biofilms are more resistant to detergents and biocides than planktonic bacteria. And biofilms constitute a protected mode of growth that allows survival in a hostile environment, including low pH, high salt concentrations, and low temperature (Costerton et al., 1999; Buchanan et al., 2017). The ability of *L. monocytogenes* to form biofilms can considerably enhance the stress tolerance and, thus, increases the persistence in a hostile environment (Vázquez-Sánchez et al., 2017).

Thymoquinone (C_10_H_12_O_2_, TQ), also called 2-isopropyl-5-methyl-1,4-benzoquinone, is the major bioactive component (27.8–57.0%) of the volatile oil isolated from *Nigella sativa* seeds (black cumin) (Harzallah et al., 2011; Zhang et al., 2018). And TQ has been proved have a very low degree of toxicity of both mice and cells (Badary et al., 1998; Harzallah et al., 2011). The LD_50_ value of TQ ranged from 1,520—3,770 mg/kg after acute oral administration of mice (Badary et al., 1998) and the IC_50_ value of TQ on Hep-2 cell line was 19.25 ± 1.6 μg/mL (Harzallah et al., 2011). In addition to this, TQ reportedly has anti-oxidant (Burits and Bucar, 2000), anti-inflammatory (Woo et al., 2012), anticancer, and antimicrobial (Forouzanfar et al., 2014) effects. Goel and Mishra (2018) found that TQ has promising antibacterial activity against *Escherichia coli, Pseudomonas aeruginosa, Bacillus subtilis*, and *Staphylococcus aureus*, and that antimicrobial activity appears to be mediated through the generation of reactive oxygen species, leading to oxidative stress and cell death. A recent report (Sharma et al., 2016) showed that TQ has synergistic antimicrobial activity with other essential oil components (cinnamaldehyde, thymol, and eugenol) against both planktonic and biofilm-associated *Staphylococcus epidermidis*. Shi et al. (2015) also demonstrated that TQ shows antimicrobial activity against *Cronobacter sakazakii* in reconstituted infant formula.

Despite previous reports confirming the antimicrobial effects of TQ against various pathogens, there are no studies on the antimicrobial activity of TQ against *L. monocytogenes* or on its effects on biofilm formation, hemolysin production, or host-cell attachment-invasion. Therefore, in the current study, the minimum inhibitory concentrations (MICs) of TQ against several *L. monocytogenes* strains were determined to evaluate its antimicrobial activity. Changes in motility, biofilm formation, hemolysin secretion, and attachment-invasion in host cells were also analyzed to determine the effects of sub-inhibitory concentrations (SICs) of TQ on the pathogenesis of *L. monocytogenes*. Finally, the transcription of seven genes related to motility, biofilm formation, hemolysin secretion, adhesion, and invasion were examined by real-time quantitative polymerase chain reaction (RT-qPCR) analysis.

## Materials and Methods

### Reagents

TQ (CAS: 490-91-5) was obtained from Tokyo Chemical Industry Co. (Tokyo, Japan) at a high-performance liquid chromatography purity of at least 99%. Stock solutions of TQ were prepared in 0.1% (v/v) dimethyl sulfoxide (DMSO) as described previously (Shi et al., 2015). All other chemicals were of analytical grade.

### Bacterial Strains and Growth Conditions

*L. monocytogenes* strains ATCC 19115 and ATCC 15313 were purchased from the American Type Culture Collection (ATCC, Manassas, VA, USA). Other *L. monocytogenes* strains (A17, A24, B9, B19, C6, and C34) were originally isolated from raw chicken meat, infant food, and ready-to-eat meals (Table 1) in Shaanxi Province, China (Zhang et al., 2013). In preparation for assays, stock cultures of bacterial strains, which were stored in 20% (v/v) glycerol at −80°C, were streaked onto tryptone soya agar (TSA) and cultured at 37°C for 30 h. A loopful of each strain was then inoculated into 30 mL of tryptic soy broth (TSB) and cultured for 18 h at 37°C with shaking (130 rpm). Following centrifugation at 8,000 × g for 5 min at 4°C, bacterial cells were washed with phosphate-buffered saline (PBS, pH = 7.2) and re-suspended in fresh PBS to achieve an optical density at 600 nm (OD_600_) of 0.5 (~10^9^ colony-forming units (CFU)/mL). All strains were used in MICs assays. *L. monocytogenes* ATCC 19115 contains phenotypic and genotypic characteristics of *Listeria*, such as the ability of biofilm formation (Winkelstroter et al., 2015), swimming and clustering, hemolysin secretion (Wang et al., 2015), and attachment-invasion of host cells (Jaradat and Bhunia, 2003). Thus, *L. monocytogenes* ATCC 19115 was selected for further experiments.

**Table 1:** Minimum inhibitory concentrations (MICs) of TQ against several *Listeria monocytogenes* strains.

**Strains**	**Origin**	**Serotypes**	**MICs (μg/mL)**
ATCC 19115	Human	4b	12.50
ATCC 15313	Rabbit	1/2a	6.25
A17	Raw chicken	1/2a	6.25
A24	Raw chicken	1/2b	12.50
B9	Infant foods	4e	12.50
B19	Infant foods	4b	12.50
C6	Ready-to-eat foods	4b	12.50
C34	Ready-to-eat foods	1/2b	6.25

### Minimum Inhibitory Concentrations Assay

MICs were determined by serial microbroth dilution according to the method of Chen et al. (2017), with some modifications. Briefly, bacterial cultures were diluted to ~10^5^ CFU/mL in TSB, and 100-μL aliquots of each diluted culture were separately transferred into the wells of a 96-well microtiter plate. An equal volume of TQ solution dissolved in TSB supplemented with 0.1% (v/v) DMSO was added each well. The final concentrations of TQ were 0 (control), 3.12, 6.25, 12.5, 25, 50, 100, and 200 μg/mL. Ampicillin (100 μg/mL) was used as a positive control, while TSB supplemented with 0.1% (v/v) DMSO was used as a negative control. Samples were incubated at 37°C for 24 h. The MIC was defined as the lowest concentration of TQ resulting in a difference in OD_630_ of <0.05 between the readings taken before and after incubation of the test strain.

### Sub-inhibitory Concentrations Assay

The SICs (concentrations not inhibiting growth) of TQ against *L. monocytogenes* strain ATCC 19115 were determined by broth dilution (Johny et al., 2010), with some modifications. Briefly, bacterial culture was diluted to ~10^7^ CFU/mL in TSB. Aliquots (100 μL) of the bacterial suspension were added to the wells of a 96-well plate. Then, an equal volume of TQ solution (100 μL) prepared in TSB was added to each well to achieve final TQ concentrations of 25, 12.5, 6.25, 3.12, 1.56, 0.78, 0.39, and 0 μg/mL. The bacterial cell density at 600 nm (OD_600_) was measured every 1 h by using the automated Bioscreen C system (Labsystems, Helsinki, Finland). And the growth curve was drawn after 24 h of incubation (at 37°C). The three highest concentrations that exhibited no significant inhibitory effect on the growth of *L. monocytogenes* strain ATCC 19115 were selected as SICs for the following assays.

### Swimming Assay

Swimming assays were performed as described by Li et al. (2014), with some modifications. Semi-solid agar medium was prepared by the addition of 0.3% (w/v) agar to 20 mL of Luria-Bertani broth (LB) (25 g/L). TQ was added to the warm medium (45°C) to obtain final concentrations of 1.56, 0.78, 0.39, and 0 μg/mL. Medium without TQ was used as a control. After drying plates for 30 min at 25°C, 5 μL of *L. monocytogenes* ATCC 19115 (~10^9^ CFU/mL) were inoculated onto the surface of the semi-solid agar. Plates were incubated at 37°C for 24 h, and the size of the swimming area in the presence or absence of TQ was calculated using Image J.

### Biofilm Formation Assays

#### Inhibition of Specific Biofilm Formation (SBF)

Assays to examine the inhibition of *L. monocytogenes* SBF were performed according to the method described by Shi et al. (2017a), with minor modifications. Briefly, *L. monocytogenes* ATCC 19115 was cultured as described in 2.2. TQ solution was prepared in the wells of 96-well plates to final concentrations of 0, 0.39, 0.78, and 1.56 μg/mL. The plates were then incubated at 25°C, 37°C, or 12°C for 3, 5, or 7 days. LB broth without bacterial suspension or TQ was used as a blank control. OD_630_ measurements were collected at each time point as a measure of cell growth. At the end of the culture period, wells were stained with 1% (w/v) crystal violet for 20 min at room temperature. The crystal violet dye solution was discarded, and the wells were washed three times with 350 μL of sterile distilled water. After air drying, the wells were decolorized with 250 μL of 33% (v/v) glacial acetic acid and incubated at room temperature with shaking (100 rpm) for 20 min. OD_570_ measurements were then collected for each well to reflect the amount of biofilm formed. SBF was calculated using the following formula: SBF = OD_570_/OD_630_ (Niu and Gilbert, 2004).

#### Scanning Electron Microscopy (SEM) Examination of Biofilm Structure

SEM assays were carried out as previously described (Shi et al., 2015), with some modifications. Bacterial culture suspension and TQ dilutions were prepared as described in 2.6.1. After mixing TQ with bacterial suspension to obtain final TQ concentrations of 0, 0.39, 0.78, and 1.56 μg/mL, the mixtures were added to the wells of a 24-well plate containing sterile glass coverslips (diameter, 10 mm). Plates were then incubated for 3 days at 37°C to allow biofilm formation. After washing twice with PBS, the slides were submerged in 2 mL of 2.5% (v/v) glutaraldehyde and incubated at 4°C for 10 h to fix bacterial cells, followed by washing with PBS. Slides were then dehydrated using an ethanol-water dilution series (30, 50, 70, 80, 90, and 100%), with incubations of 10 min per concentration. Following dehydration, the slides were thoroughly air-dried and immediately sputter-coated with gold under vacuum, followed by observation using a field emission scanning electron microscope (S-4800; Hitachi, Tokyo, Japan).

### Adhesion and Invasion Assays

The effect of TQ on the ability of *L. monocytogenes* to adhere to and invade host cells was investigated as previously described (Moroni et al., 2006). Human colon adenocarcinoma cell line Caco-2 was cultured as previously reported (Fan et al., 2018). For the assays, trypsin-treated Caco-2 cells were seeded into a 24-well tissue culture plate (10^5^ cells per well) and incubated for 18 h under 5% CO_2_ at 37°C. *L. monocytogenes* was grown to mid-log phase with or without SICs of TQ, then harvested and washed twice in PBS and re-suspended in Dulbecco's Modified Eagle Medium (DMEM) at a final concentration of 10^7^ CFU/mL. Caco-2 cell monolayers were then rinsed twice with PBS, and bacterial suspension was added to each well to an MOI = 100. Plates were then centrifuged at 600 × g for 5 min and incubated at 37°C in a humidified, 5% CO_2_ incubator for 2 h.

For adhesion assays, cells with adherent bacteria were rinsed after centrifugation (600 × g, 5 min), before being lysed by incubation with 1 mL of 0.1% (v/v) Triton X-100 at 4°C for 20 min. The number of viable adherent *L. monocytogenes* cells was determined by plating serial dilutions of the lysed cells on TSA plates and counting the resulting cells following incubation at 37°C for 36 h.

For invasion assays, the cell monolayers were incubated for 2 h following inoculation, rinsed three times with PBS, and then incubated for a further 45 min following the addition of DMEM supplemented with gentamicin (100 μg/mL) to kill the extracellular bacteria. Finally, the cells were washed three times with PBS before being lysed and plated as described in the adhesion assay. Invasive bacterial cell counts were expressed as a percentage relative to that of the control.

### Hemolysis Assay

Hemolytic activity was measured as described by Liu et al. (2016) to assess the effects of TQ on LLO secretion by *L. monocytogenes*. Briefly, *L. monocytogenes* ATCC 19115 was cultured in brain heart infusion (BHI) broth supplemented with SICs of TQ (1.56, 0.78, or 0.39 μg/mL) then incubated as described in 2.2. After centrifugation (5,500 × g, 10 min, 4°C), aliquots (100 μL) of the supernatant were mixed with 100 μL of freshly-washed sheep red blood cells and 1 mL of hemolysin buffer (0.145 mol/L NaCl, 0.02 mol/L CaCl_2_). The mixtures were incubated for 30 min before being centrifuged at 5,500 × g for 10 min at 4°C, then recorded the OD_450_ of each mixture. A mixture consisting of 100 μL of culture medium and 1 mL of hemolysin buffer served as the negative control (0% hemolysis), while a mixture of 100 μL of 1% Triton X-100 and 1 mL of 10% sheep red blood cells was used as the positive control (100% hemolysis). Percent hemolysis was estimated according to the following formula: hemolysis (%) = (OD_s_-OD_n_)/(OD_p_-OD_n_) × 100, where OD_s_, OD_n_, and OD_p_ are the absorbance values of the sample, negative control, and positive control, respectively (Du et al., 2018).

### Isolation of RNA and RT-qPCR Analysis

RT-qPCR analysis was conducted to determine the effects of TQ on the transcription of genes associated with motility, biofilm formation, hemolysin secretion, and adhesion and invasion of host cells, as previously reported (Shi et al., 2017b). In brief, *L. monocytogenes* ATCC 19115 was cultured and harvested as described in 2.7. Total RNA was extracted using an RNAprep Pure Bacteria Kit (Tiangen, Beijing, China) according to the manufacturer's instructions, before being reverse-transcribed into cDNA using a PrimeScript RT Reagent Kit (Takara, Kyoto, Japan). First-strand cDNA was synthesized from 450 ng of each RNA sample in a 10-μL reaction volume. RT-qPCR assays were performed in a 25-μL reaction volume using SYBR Premix Ex Taq II (Takara). Reaction components and cycling conditions were as described previously (Shi et al., 2017b). All samples were examined in triplicate and normalized to the expression of the endogenous control gene (16S rRNA). Gene transcription levels were determined using the 2^−ΔΔ*Ct*^ method. Primer sequences used for RT-qPCR analysis are provided in Table 2.

**Table 2:** Primers used in this study.

**Genes**	**Primer**	**Sequence (5^**′**^-3^**′**^)**
16S rRNA	Forward	ACCGTCAAGGGACAAGCA
	Reverse	GGGAGGCAGCAGTAGGGA
*agrA*	Forward	ATGAAGCAAGCGGAAGAAC
	Reverse	TACGACCTGTGACAACGATAAA
*flaA*	Forward	CTGGTATGAGTCGCCTTAG
	Reverse	CATTTGCGGTGTTTGGTTTG
*hly*	Forward	AACCAGATGTTCTCCCTGTA
	Reverse	CACTGTAAGCCATTTCGTCA
*inlB*	Forward	AAGCAMGATTTCATGGGAGAGT
	Reverse	TTACCGTTCCATCAACATCATAACTT
*plcB*	Forward	CAGGCTACCACTGTGCATATGAA
	Reverse	CCATGTCTTCYGTTGCTTGATAATTG
*prfA*	Forward	ATGAACGCTCAAGCAGAAGA
	Reverse	CGAAAGCACCTTTGTAGTATTG
*sigB*	Forward	GATGATGGATTTGAACGTGTGAA
	Reverse	CGCTCATCTAAAACAGGGAGAAC

*Y indicates C or T*.

*M indicates A or C*.

### Statistical Analysis

All samples were examined in triplicate and all results are expressed as the mean ± standard error of the mean. One-way analysis of variance was performed to identify significant differences among groups. *Post-hoc* Turkey's multiple comparison tests and least significant difference tests were used to evaluate significant differences. All analyses were conducted using SPSS version 19.0 (SPSS Inc., Chicago, IL). Significant differences are indicated by ^*^P < 0.05, and ^**^P < 0.01.

## Results

### MICs and SICs of TQ

The MICs of TQ against the eight tested *L. monocytogenes* strains are listed in Table 1. Overall, MICs ranged from 6.25—12.50 μg/mL, with strains ATCC 15313, A17, and C34 showing the greatest sensitivity to TQ (MIC = 6.25 μg/mL).

**Figure 1:**
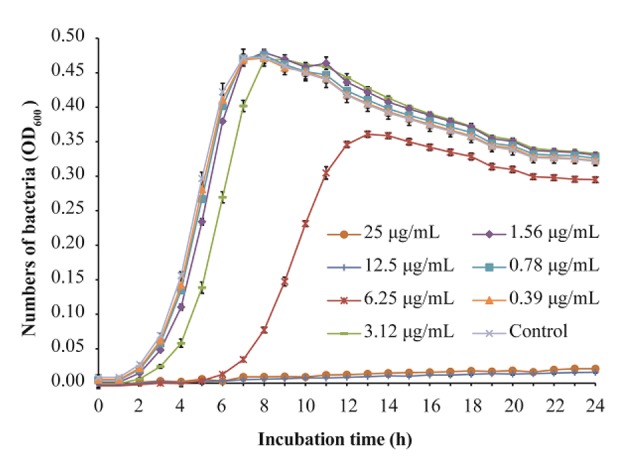
Growth of *Listeria monocytogenes* ATCC 19115 culture in tryptic soy broth supplemented with various concentrations of thymoquinone (TQ). Bars indicate the standard deviation (*n* = 6).

*L. monocytogenes* strain ATCC 19115 was selected to use in further analyses. The MIC of TQ against *L. monocytogenes* ATCC 19115 was 12.50 μg/mL. The growth of strain ATCC 19115 in TSB supplemented with various concentrations of TQ is shown in Figure 1. Concentrations of TQ below 1.56 μg/mL exhibited no inhibitory effect against ATCC 19115 (Figure 1). As such, 0.39 (1/32MIC) μg/mL, 0.78 (1/16MIC) μg/mL, and 1.56 (1/8MIC) μg/mL were chosen as the SICs for further experiments.

### Swimming Motility

**Figure 2:**
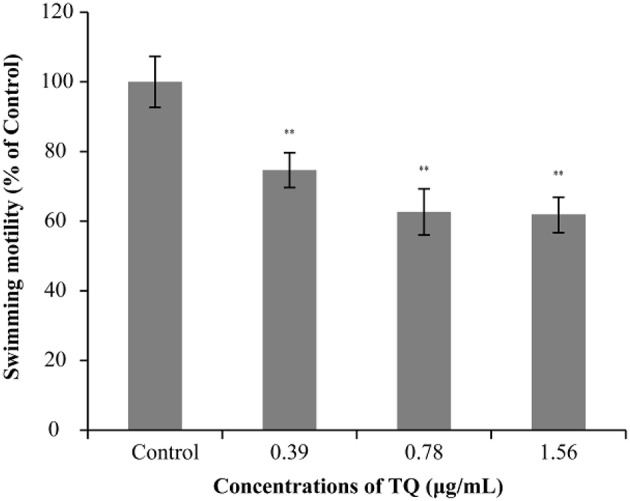
Effect of thymoquinone (TQ) on swimming motility of *Listeria monocytogenes* ATCC 19115. Percentage of the size of the swimming area relative to the control group is presented as the mean ± the standard deviation of three independent experiments. Bars indicate the standard deviation (*n* = 3). ^**^P < 0.01.

The effect of TQ on the swimming motility of *L. monocytogenes* ATCC 19115 is shown in Figure 2. The swimming motility of strain ATCC 19115 was significantly inhibited by TQ at its SICs (0.39, 0.78, and 1.56 μg/mL). The size of the swimming area of control is 7.03 ± 0.77 cm^2^. At TQ concentrations of 0.39, 0.78, and 1.56 μg/mL, the swimming motility of cells in the TQ treatment group was decreased (*P* < 0.01) by 25.35, 37.33, and 38.02%, respectively, compared with the control.

### Effects of TQ on Biofilm Formation by *L. monocytogenes* ATCC 19115

**Figure 3:**
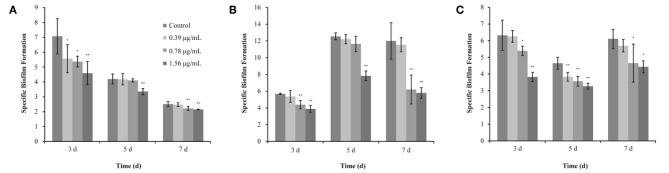
Inhibitory effects of different concentrations of thymoquinone (TQ) on biofilm formation by *Listeria monocytogenes* ATCC 19115 at 37°C **(A)**, 25°C **(B)**, and 12°C **(C)**. Bars indicate the standard deviation (*n* = 3). ^*^P < 0.05; ^**^P < 0.01.

The anti-biofilm efficacy of TQ was investigated using *L. monocytogenes* ATCC 19115 incubated at 37, 25, or 12°C for 3, 5, or 7 days. As shown in Figure 3, TQ at concentration of 1.56 μg/mL, TQ significantly (*P* < 0.05) reduced biofilm formation compared with the control at each of the incubation condition.

The SBF index (OD_570_/OD_630_) of *L. monocytogenes* ATCC 19115 (without TQ treatment) was 7.07 ± 1.19, 4.20 ± 0.33, and 2.51 ± 0.16, after incubation at 37°C for 3, 5, and 7 days, respectively (Figure 3A). TQ at concentrations of 0.39, 0.78, and 1.56 μg/mL was significantly decreased the SBF index of *L. monocytogenes* ATCC 19115 to 5.56 ± 0.94 (*P* < 0.05), 5.37 ± 0.36 (*P* < 0.05), and 4.59 ± 0.78 (*P* < 0.01), respectively, following incubation at 37°C for 3 days (Figure 3A). Following incubation for 5 days at 37°C, the SBF index of *L. monocytogenes* ATCC 19115 was significantly reduced to 3.35 ± 0.20 (*P* < 0.01) only in the presence of TQ at concentrations of 1.56 μg/mL (Figure 3A). Addition of TQ at 0.78 and 1.56 μg/mL caused the SBF index of *L. monocytogenes* ATCC 19115 reductions to 2.22 ± 0.13 and 2.15 ± 0.02 (*P* < 0.01), while TQ at 0.39 μg/mL had no significant anti-biofilm efficacy (*P* > 0.05) for cells following growth at 37°C for 7 days (Figure 3A).

The SBF index of *L. monocytogenes* ATCC 19115 following growth at 25°C for 3, 5, and 7 days in control group was 5.67 ± 0.08, 12.55 ± 0.42, and 12.00 ± 2.18, respectively (Figure 3B). For *L. monocytogenes* ATCC 19115 incubated at 25°C (Figure 3B), the SBF index of cells treated with 1.56 μg/mL TQ was decreased to 3.88 ± 0.40, 7.83 ± 0.57, and 5.79 ± 0.63, after 3, 5, and 7 days, respectively. TQ at 0.78 μg/mL reduced (*P* < 0.01) the initial SBF index to 4.39 ± 0.49 and 6.20 ± 1.73 after 3, and 7 days at 25°C, respectively, while TQ at 0.78 μg/mL was not significantly reduced biofilm formation of cells following growth at 25°C for 5 days (Figure 3B). With SIC of 0.39 μg/mL, TQ showed no significant anti-biofilm effect (*P* > 0.05) for *L. monocytogenes* ATCC 19115, followed by incubation for 3, 5, and 7 days at 25°C (Figure 3B).

As shown in Figure 3C, the SBF index of *L. monocytogenes* ATCC 19115 following growth at 12°C for 3, 5, and 7 days in control group was 6.35 ± 0.90, 4.65 ± 0.36, and 6.11 ± 0.58, respectively. After incubation at 12°C for 3 days with the presence of TQ at concentrations of 0.39, 0.78, and 1.56 μg/mL, the SBF index of *L. monocytogenes* ATCC 19115 was decreased to 6.26 ± 0.35, 5.39 ± 0.28 (*P* < 0.05), and 3.83 ± 0.27 (*P* < 0.01), respectively (Figure 3C). TQ significantly (*P* < 0.01) reduced biofilm formation of *L. monocytogenes* ATCC 19115, following growth at 12°C for 5 days, by 17.42, 23.24, 29.52% compared with the control at TQ concentrations of 0.39, 0.78, and 1.56 μg/mL, respectively (Figure 3C). Compared with the control, the biofilm formation of cells treated with TQ at concentrations of 0.78 and 1.56 μg/mL was inhibited (*P* < 0.05) by 23.83 and 27.41%, respectively, after 7 days of incubation at 12°C (Figure 3C). With SIC of 0.39 μg/mL, TQ showed no significant anti-biofilm effect (*P* > 0.05) for *L. monocytogenes* ATCC 19115, followed by incubation for 7 days at 12°C (Figure 3C).

### SEM Analysis of Biofilm Structure

**Figure 4:**
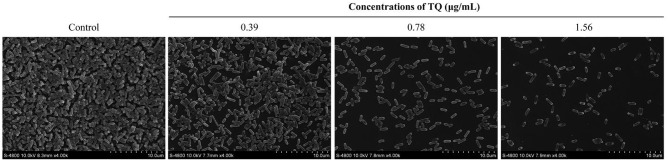
Field emission scanning electron microscope images showing the effects of different concentrations of thymoquinone (TQ) on biofilm structure following incubation of *Listeria monocytogenes* ATCC 19115 at 37°C for 3 days.

The effect of TQ on *L. monocytogenes* ATCC 19115 biofilm structure was examined using SEM (Figure 4). SEM images showed that *L. monocytogenes* ATCC 19115 exhibited a large-scale coral-like three-dimensional structure, with a high degree of stacking in the control culture. Following increases in the concentration of TQ, the number of bacteria attached to the surface gradually decreased, and the biofilm itself became thinner, looser, and less uniform. Therefore, SEM analysis suggested that TQ destroys the stereostructure of *L. monocytogenes* ATCC 19115 biofilms.

### Adhesion to and Invasion of Caco-2 Cells

**Figure 5:**
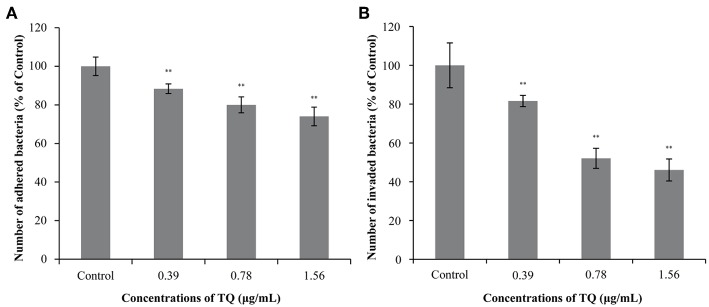
Effects of thymoquinone (TQ) on the ability of *Listeria monocytogenes* ATCC 19115 to adhere to **(A)** and invade **(B)** Caco-2 cells. Bars indicate the standard deviation (*n* = 3). ^**^P < 0.01.

Figure 5 shows the effects of TQ on the adhesion to and invasion of Caco-2 cells by *L. monocytogenes* ATCC 19115. At TQ concentrations of 0.39, 0.78, and 1.56 μg/mL, the adhesion rate of *L. monocytogenes* ATCC 19115 was reduced to 88.36, 80.03, and 74.02% of that of the control, respectively (Figure 5A). The SICs of TQ also reduced the invasion rate of *L. monocytogenes* ATCC 19115, with rates of 81.59, 52.06, and 46.08% of those of the control at the respective TQ concentrations (Figure 5B). All decreases were significant (*P* < 0.01), confirming that TQ effectively inhibited the ability of *L. monocytogenes* ATCC 19115 to adhere to and invade Caco-2 cells.

### LLO Secretion

**Figure 6:**
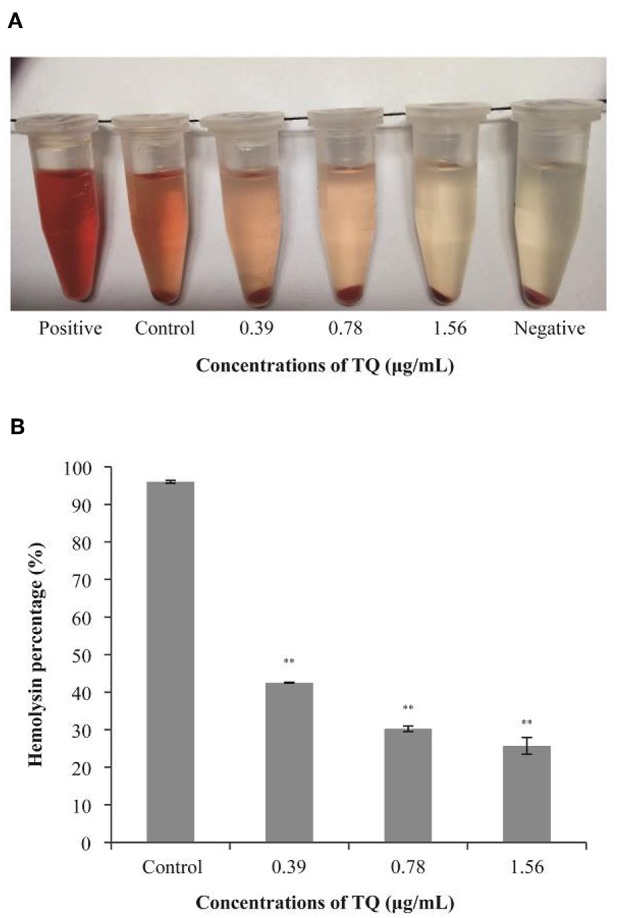
Hemolysis of sheep red blood cells by *Listeria monocytogenes* ATCC 19115 culture supernatants in the absence or presence of sub-inhibitory concentrations of thymoquinone (TQ). Qualitative **(A)** and quantitative **(B)** analysis results are shown. Bars indicate the standard deviation (*n* = 5). ^**^P < 0.01.

Hemolysis assays confirmed that the LLO secretion rates of *L. monocytogenes* ATCC 19115 preincubated with SICs of TQ were significantly lower than that of the positive control (Figure 6A). As shown in Figure 6B, in the presence of TQ at concentrations of 0.39, 0.78, and 1.56 μg/mL, LLO secretion rates were reduced to 42.52, 30.25, and 25.69%, respectively, of those of the control. The results indicated that TQ inhibits the secretion of LLO by *L. monocytogenes* ATCC 19115.

### Effect of TQ on the Transcription of Genes Related to Motility, Biofilm Formation, Hemolysin Secretion, and Adhesion and Invasion

**Figure 7:**
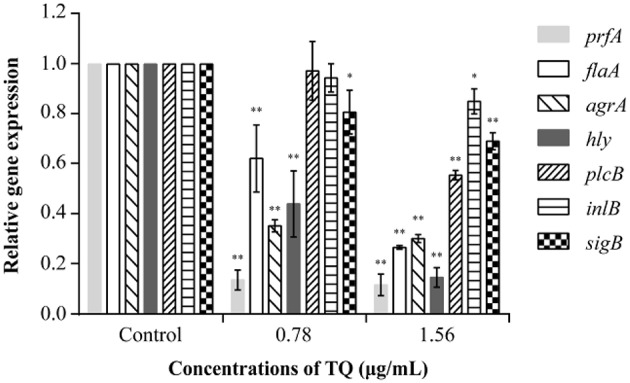
Effect of thymoquinone (TQ) on the transcription of *Listeria monocytogenes* ATCC 19115 virulence genes. Bars indicate the standard deviation (*n* = 3). ^*^P < 0.05; ^**^P < 0.01.

RT-qPCR analysis revealed that at SICs, TQ down-regulated the expression of seven *L. monocytogenes* ATCC 19115 genes associated with motility, biofilm formation, hemolysin secretion, and attachment-invasion in host cells in a dose-dependent manner (Figure 7). TQ significantly (*P* < 0.05) down-regulated the transcription of *prfA* (transcriptional regulator-encoding gene), *flaA* (flagellin gene), *agrA* [quorum sensing (QS) response regulator], *hly* (listeriolysin O gene), and *sigB* (stress response factor) to various degrees. The transcription of *plcB* (phosphatidylcholine phospholipase-encoding gene) and *inlB* (internalization protein regulatory gene), which are both related to adhesion and invasion of host cells, was also down-regulated at a TQ concentration of 0.78 μg/mL, but the decrease was not significant.

## Discussion

As a common food-borne pathogen, *L. monocytogenes* can cross the intestinal, blood-brain, and placental barriers to cause a variety of serious diseases, even death. The ability of *L. monocytogenes* to develop resistance to antimicrobial agents has forced researchers to look for novel and effective bacteriostatic agents to inhibit biofilm formation, hemolysin production, and attachment-invasion in host cells, and to explore the resistance mechanisms of *L. monocytogenes*.

In the current study, TQ effectively inhibited the growth of eight *L. monocytogenes* strains from both clinical and environmental (ready-to-eat foods) sources, which may suggest universal efficacy against *L. monocytogenes*. To confirm this, a greater number of *L. monocytogenes* strains from various clinical and environmental sources should be tested. Fan et al. (2018) reported that the MICs of coenzyme Q0 against *L. monocytogenes* strains ranged from 25—50 μg/mL, while the MICs of thymol and lactobionic acid against *L. monocytogenes* were 0.25 and 10 mg/mL, respectively (Chen and Zhong, 2017). Using the broth microdilution method, the MIC of epigallocatechin-gallate against *L. monocytogenes* was determined to be 200 μg/mL (Du et al., 2018). Further, anthocyanins from wild blueberries were also studied for their inhibitory effects on *L. monocytogenes*, showing a MIC of 0.27 mg/mL (Sun et al., 2018). MIC determination assays conducted in the current study revealed the potent *in vitro* antibacterial properties of TQ (MICs of 6.25 or 12.50 μg/mL) against all eight tested *L. monocytogenes* strains (Table 1). Based on the previous reports, it appears that TQ is by far the most effective natural product for inhibiting *L. monocytogenes*.

Based on the determined SICs, we explored the inhibitory effect of TQ on *L. monocytogenes* biofilm formation. The results showed that TQ significantly reduces biofilm formation (Figure 3) and affects biofilm stereostructure (Figure 4) when bacteria are cultured at 12, 25, or 37°C. Similarly, cinnamaldehyde, and carvacrol have good inhibitory effects against biofilm formation by *L. monocytogenes*, also affecting the stereostructure, at 4, 25, and 37°C (Upadhyay et al., 2013), while Du et al. (2018) found that epigallocatechin-gallate significantly reduces biofilm formation at 15, 30, and 37°C. In addition, the results of RT-qPCR assays (Figure 7) carried out in the current study illustrated that SICs of TQ significantly (*P* < 0.01) decrease the transcription of *agrA*, which encodes the QS response regulator in *L. monocytogenes* (Du et al., 2018). Numerous studies indicate that QS is linked to the synthesis of substances involved in biofilm formation, including exopolysaccharides and polysaccharide intercellular adhesin (Musthafa et al., 2010; Di Cagno et al., 2011). Therefore, we speculate that TQ inhibits biofilm formation by affecting QS in *L. monocytogenes*, thereby weakening its resistance to adverse environmental conditions.

Some reports indicate that flagellar motility plays an important role in biofilm formation by *L. monocytogenes* because bacteria first move to the surface of host cells before adhering and invading (Lemon et al., 2007; Fuente-Núñez et al., 2012). In this study, we examined the mobility of *L. monocytogenes* cultured at 37°C in the presence of TQ. The results showed that bacterial motility was significantly inhibited by TQ in a concentration-dependent manner (Figure 2). Similarly, Du et al. (2018) demonstrated that SICs of epigallocatechin-gallate significantly inhibited the motility of *L. monocytogenes*. *FlaA* is involved in the regulation of flagellin synthesis and expression and plays a key role in *L. monocytogenes* movement (Williams et al., 2005). RT-qPCR analysis showed that *flaA* transcription was significantly decreased (Figure 7) in TQ-treated *L. monocytogenes* cells compared with the control; thus, we speculate that TQ may inhibit the activity of *L. monocytogenes* by regulating the synthesis of flagellin.

To enhance its own survival and proliferation, *L. monocytogenes* adheres to and then invades host cells, ultimately leading to tissue lesions. This process is recognized as an important pathway for bacterial infection (Caplan and Mateescu, 2013). The first step of *L. monocytogenes* infection is attaching to and invading the intestinal epithelial barrier, followed by systemic spread to other tissues, including the central nervous system and placenta (Parida et al., 1998). In the current study, both the adhesion and invasion rates of *L. monocytogenes* ATCC 19115 were significantly reduced following preincubation with TQ (Figure 5). In both cases, the decreases followed a concentration-dependent trend. Fan et al. (2018) also reported that coenzyme Q0 inhibited the attachment and invasion of *L. monocytogenes* CMCC 54004, while Xu et al. (2015) found that the tannin-rich fraction from pomegranate rind homogenate remarkably reduced the ability of *L. monocytogenes* to adhere to and invade Caco-2 cells in a dose-dependent manner. Upadhyay et al. (2012) showed that trans-cinnamaldehyde, carvacrol, and thymol down-regulated the transcription of genes encoding *Listeria* adhesion (*lmo1634, lmo1666*, and *lmo1847*) and invasion (*iap* and *lmo1076*) proteins, resulting in decreased host-cell adherence and invasion. RT-qPCR analysis conducted in the current study showed that SICs of TQ significantly (*P* < 0.05) down-regulated the transcription of genes associated with adhesion and invasion (Figure 7) such as *sigB* and also decreased the transcription of *inlB*, an invasion associated gene. Therefore, we speculate that TQ regulates the ability of *L. monocytogenes* to adhere to and invade host cells by modulating the activity of regulatory proteins.

LLO is a hemolysin produced by *L. monocytogenes*. It is an important pathogenicity factor, forming pores in the phagocytic cell membrane that allow bacteria to escape from within the phagosome into the cytosol, where they proliferate (Hamon et al., 2012). In this study, sheep red blood cells were used to explore the effects of TQ on the secretion of LLO by *L. monocytogenes* ATCC 19115. The results showed that TQ significantly (*P* < 0.01) inhibits the secretion of LLO (Figure 6) by decreasing the transcription of LLO secretion gene *hly* (Figure 7). This finding was in accordance with the results of a previous study by Liu et al. (2016), who showed that tea tree oil inhibited the hemolysis activity of α-hemolysin, thereby reducing the secretion of LLO by *L. monocytogenes*. Zhou et al. (2017) also investigated the protective effects of curcumin against *L. monocytogenes* infection by targeting LLO. Hemolytic activity assays and cytotoxicity tests revealed that treatment of infected macrophages with curcumin leads to a decrease in LLO-mediated bacterial phagosome escape and limits the intracellular growth of *L. monocytogenes*. In our future studies, inhibition effect of TQ on virulence factors of other strains of *L. monocytogenes* will be explored.

## Conclusions

In summary, the MICs of TQ against several *L. monocytogenes* strains were determined for the first time. With MICs ranging from 6.25—12.5 μg/mL, TQ exhibited significant antimicrobial activity against the tested *L. monocytogenes* strains. SICs of TQ effectively inhibited swimming motility and reduced biofilm formation. Furthermore, TQ decreased the ability of *L. monocytogenes* to adhere to and invade Caco-2 cells, and reduced the secretion of hemolysin LLO. RT-qPCR analyses confirmed that TQ down-regulates the transcription of genes associated with swimming motility, biofilm formation, hemolysin secretion, and host-cell adhesion and invasion. Therefore, this study suggests that TQ has the potential to be used as an alternative or supplemental strategy to mitigate the infections caused by *L. monocytogenes*. In our future research, an animal study of TQ on virulence factors of *Listeria* and the molecular mechanism *in vitro* will be explored.

## Data Availability

All datasets generated for this study are included in the manuscript and the supplementary files.

## Author Contributions

CS and XM conceived and designed the experiments. HL, YZ, and DG performed the experiments. YX analyzed the data. XX contributed reagents, materials, and analysis tools. XM and HL wrote the manuscript.

### Conflict of Interest Statement

The authors declare that the research was conducted in the absence of any commercial or financial relationships that could be construed as a potential conflict of interest.
